# Evolving Trends and Collaborative Networks in Postoperative Delirium Research: A Two‐Decade Bibliometric Analysis

**DOI:** 10.1002/brb3.71558

**Published:** 2026-06-22

**Authors:** Kehan Wang, Cheng Xu, Yuan Zhu, Quanhong Zhou, Tao Xu

**Affiliations:** ^1^ Department of Anesthesiology Shanghai Sixth People's Hospital Affiliated to Shanghai Jiao Tong University School of Medicine Shanghai China; ^2^ Department of Critical Care Medicine Shanghai Sixth People's Hospital Affiliated to Shanghai Jiao Tong University School of Medicine Shanghai China

**Keywords:** bibliometric analysis, postoperative delirium, visualization

## Abstract

**Objective:**

Postoperative delirium (POD) remains a prevalent, complex complication in hospitalized older adults, yet its multifactorial pathogenesis and optimal management strategies elude definitive characterization. This study aimed to conduct a comprehensive bibliometric analysis of POD research over the past two decades to identify publication trends, collaborative networks, and major thematic areas.

**Methods:**

We conducted a systematic bibliometric analysis of English‐language POD publications in the Web of Science Core Collection from January 2004 to August 2024. A rigorous multistage screening process was implemented using advanced analytical tools, including CiteSpace and VOSviewer.

**Results:**

The analysis encompassed 1937 articles from 65 countries, with annual publication output accelerating significantly after 2019. China ranked first in publication volume, whereas the United States had the highest citation count and total link strength, indicating greater centrality in the international collaboration network. Co‐citation and keyword analyses identified four major research domains: characteristics, mechanisms, prediction, and interventions. Recent growth has concentrated in prediction‐oriented and biomarker‐related research.

**Conclusion:**

This bibliometric study elucidates the dynamic evolution of POD research, highlighting growing interest in predictive analytics and mechanistic biomarker research as potential priorities for future investigation. These findings not only inform targeted future investigations but also guide the development of interdisciplinary strategies to improve diagnostic precision and therapeutic interventions for acute neurocognitive dysfunction in older surgical populations.

## Graphical abstract

This bibliometric study analyzes 1,937 POD articles from 65 countries, with annual publications accelerating markedly after 2019. China leads in publication volume, while the US tops citations and holds stronger centrality in the international collaboration network. Four major research domains are identified: characteristics, mechanisms, prediction and interventions, with prediction and biomarkers as fast‐growing hotspots. The findings outline the field's evolution and guide interdisciplinary strategies to improve diagnosis and treatment for older surgical patients.

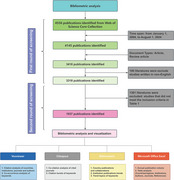

**Abbreviations**
APOE: apolipoprotein E
CAM: Confusion Assessment Method
CRP: C‐reactive protein
DSM‐5: Diagnostic and Statistical Manual of Mental Disorders, Fifth Edition
DSM‐IV: Diagnostic and Statistical Manual of Mental Disorders, Fourth Edition
EVs: extracellular vesicles
HELP: Hospital Elder Life Program
ICU: intensive care unit
IF: impact factor
IL: interleukin
LLR: log‐likelihood ratio
POD: postoperative delirium
SCIE: Science Citation Index Expanded
SSCI: Social Sciences Citation Index
TNF: tumor necrosis factor
WoSCC: Web of Science Core Collection

## 1 Introduction

Postoperative delirium (POD) represents a pervasive and formidable challenge in perioperative care, frequently identified as a major complication affecting hospitalized patients (Drews et al. 2015). Rather than a mere temporary confusional state, this syndrome reflects acute brain dysfunction characterized by fluctuating inattention and altered awareness, usually emerging 2–5 days after surgery. Epidemiology varies widely, with incidence rates fluctuating between negligible figures in stable patients undergoing minor procedures to over 50% in vulnerable populations requiring major or emergent interventions (Gleason et al. 2015; C.‐G. Wang et al. 2018; Ormseth et al. 2023). Given the rapidly aging global demographic and increasing surgical volumes, POD has evolved from a minor clinical concern into a priority for public health policy.

The deleterious sequelae of POD persist far beyond the acute perioperative phase, initiating a series of unfavorable clinical outcomes. This condition serves as a robust independent risk factor for complications, elevated in‐hospital death rates, and extended reliance on critical care services (Gleason et al. 2015; Stollings et al. 2021; Franco et al. 2001). Long‐term consequences are similarly severe, with survivors frequently experiencing enduring functional decline, lasting symptoms of posttraumatic stress, and heightened mortality risk for up to a year following discharge. Such neurocognitive impacts often accelerate cognitive deterioration and diminish quality of life, highlighting the syndrome's profound effect on patients and caregivers alike (Marcantonio 2017). Moreover, the financial burden imposed by this condition is substantial. Annual excess healthcare costs attributable to POD in the United States are estimated at approximately $32.7 billion (Gou et al. 2021). The combination of significant morbidity and economic pressure necessitates a reassessment of current research, prevention, and management paradigms.

Although studied for decades, a substantial disconnect persists between biological discoveries and their translation into effective clinical applications. The pathogenesis of POD is multifactorial, involving neuroinflammation, vascular dysfunction, and neurotransmitter dysregulation; however, an integrated mechanistic framework has not yet been established (Maldonado 2013). Though recent evidence has emerged regarding both pharmacological and nonpharmacological interventions for POD—including the potential benefits of ketamine as a neuroprotective agent and aromatherapy in reducing POD incidence in cardiac surgery—specific treatments to prevent or reverse the disorder remain lacking (Siddiqi et al. 2016; Aligholizadeh et al. 2026; Drews et al. 2026; Maroufi et al. 2024). Despite ongoing exploration of biomarkers and causality, the research landscape remains notably fragmented.

This limited progress highlights the shortcomings of conventional approaches to knowledge synthesis in a rapidly expanding field. While academic interest grows, the rapid expansion of literature generates an overwhelming volume of data, hindering the distinction between true innovation and redundancy (Nakagawa et al. 2019; Guler et al. 2016). Addressing these persistent clinical uncertainties requires a structural analysis of the scientific domain itself, rather than another narrative overview. In this context, bibliometric methods provide a useful quantitative approach for analyzing the structure and evolution of the literature. Unlike meta‐analyses that pool clinical data, this approach maps collaborations, tracks temporal trends, and reveals structural deficiencies of research output.

Accordingly, the present study aimed to apply bibliometric methods to characterize the evolution of POD research from 2004 to 2024. Specifically, we aim to review the growing global literature, identify the most influential countries, institutions, authors, and journals, map the major collaboration and intellectual networks, and define emerging hot topics and likely future directions in POD research. Through these questions, the study aims to provide a clearer structural understanding of the field and a more strategic research and clinical development plan moving forward.

## 2 Methods

### 2.1 Data Source

The Web of Science Core Collection (WoSCC) is a widely used and well‐established database for bibliometric research. Because POD spans anesthesiology, neuroscience, and biomedical science, WoSCC provides broad, multidisciplinary coverage and facilitates comprehensive literature searches. On September 1, 2024, we conducted an extensive search of the WoSCC database to gather detailed data for our bibliometric analysis of POD.

### 2.2 Search Strategy

The search was conducted in the WoSCC (Science Citation Index Expanded [SCIE] and Social Sciences Citation Index [SSCI]) on September 1, 2024, using the Topic field (TS), which searches titles, abstracts, author keywords, and Keywords Plus. The exact search query was as follows: TS = (“postoperative delirium” OR “postoperative delirium” OR “postsurgical delirium” OR “postsurgical delirium”) NOT TS = (“cognitive dysfunction” OR “POCD” OR “cognitive decline”). Filters were applied for English‐language publications published between January 1, 2004, and August 1, 2024. Document types were restricted to Articles and Review Articles. Conference abstracts, editorials, proceedings papers, early access publications, letters, corrections, and book chapters were excluded. Annual publication trends were calculated using the final indexed publication year of included records; early‐access records were excluded to avoid distorting year‐wise counts. The initial search yielded 4556 publications (Table 1).

**TABLE 1:** Search strategy of postoperative delirium.

	Search criteria	Records
Records identified through the Web of Science Core Collection database searching	TS = (“postoperative delirium” OR “postoperative delirium” OR “postsurgical delirium” OR “postsurgical delirium”) NOT TS = (“cognitive dysfunction” OR “POCD” OR “cognitive decline”)	4556
First round of screening	Time: January 1, 2004, to August 1, 2024	3318
	Languages: English	
	Document types: Articles and Review Articles	
Second round of screening (excluded literature)	Does not meet content standards (717) Does not meet quality criteria (664)	—
Remaining publications	1937 articles	1937

**FIGURE 1:**
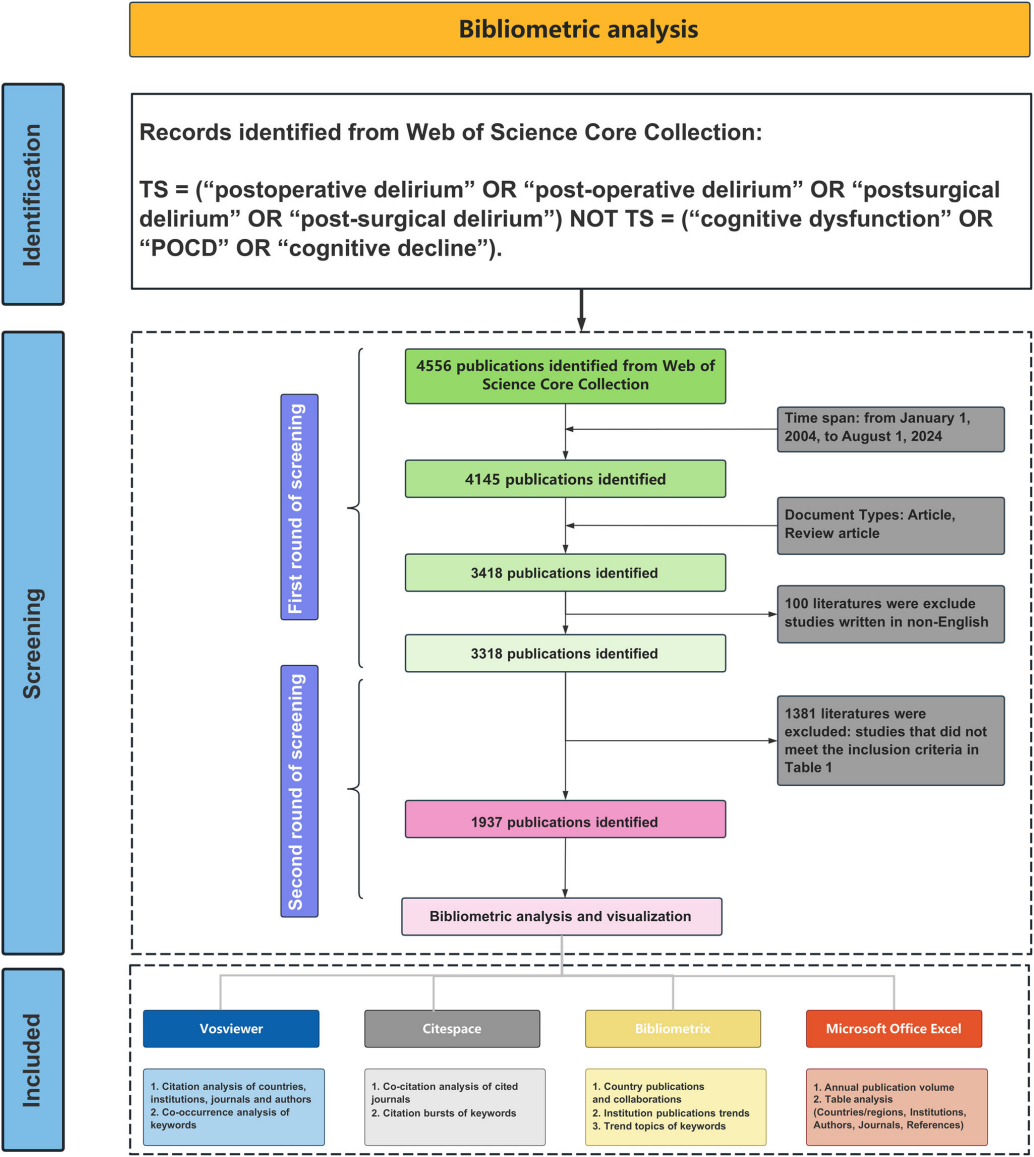
Flowchart of literature screening related to postoperative delirium.

To improve transparency of study selection, the screening process is now presented in a PRISMA‐style flow diagram (Figure 1). Records were identified from the WoSCC (*n* = 4556), filtered by time range, language, and document type (*n* = 3318), and then manually screened using predefined content and quality criteria (Table 2). During manual screening, 717 records were excluded for not meeting content criteria and 664 for not meeting quality criteria. No duplicate records were identified after CiteSpace‐based checking. Two reviewers independently screened all records, cleaned the data, and conducted cross‐validation. Disagreements were adjudicated by a blinded senior reviewer, with unresolved discrepancies settled by consensus using a predefined criteria matrix. A total of 1937 records were ultimately included in the analysis.

**TABLE 2:** Content criteria and quality criteria for the second round of manual screening.

Type	Inclusion criteria
Content criteria	The included literature should focus on postoperative delirium. Changes in other mental states, such as postoperative oversedation, metabolic encephalopathy, central cholinergic crisis, delirium‐like agitation, dementia, and postoperative cognitive dysfunction, are not considered for inclusion. The included studies are not limited to postoperative delirium but also cover its pathological and physiological mechanisms, indications for treatment, selection and comparison of therapeutic drugs and regimens, efficacy, prognosis, and health management before and after treatment.
Quality criteria	The included literature must be at least three pages long, and any documents below this standard will be excluded.^a^ Articles labeled as articles but actually summarizing reviews or meta‐analyses will be excluded. The literature must contain the basic elements of an academic paper, such as an abstract, author information, keywords, and references. Any documents lacking these elements will be excluded.

^a^ Literatures comprise meeting abstracts, brief letters to the editor, errata, or non‐peer‐reviewed commentaries.

### 2.3 Data Analysis

Bibliometric analysis was used to evaluate research trends and thematic development using VOSviewer, CiteSpace, Excel, and the Bibliometric online platform. Reproducibility depends on clear reporting of the analytic parameters used. VOSviewer facilitates detailed analysis of contributions by country, institution, and publication keywords, whereas CiteSpace is chiefly employed to classify related fields and detect burst terms. In VOSviewer, the text now explicitly states that the “Association Strength” normalization method was used for all network edge weights. In CiteSpace, we used 1‐year time slices from 2004 to 2024 and applied the log‐likelihood ratio (LLR) algorithm for noun phrase extraction and cluster labeling. Prism and Excel are utilized to generate fundamental statistical charts. The Bibliometric online platform was used to assess international collaboration patterns and to support result visualization. This study focuses on overall publication trends, national and institutional contributions, collaboration networks, publication sources, research field classification, keyword clustering, and the delineation of future research hotspots.

## 3 Results

**FIGURE 2:**
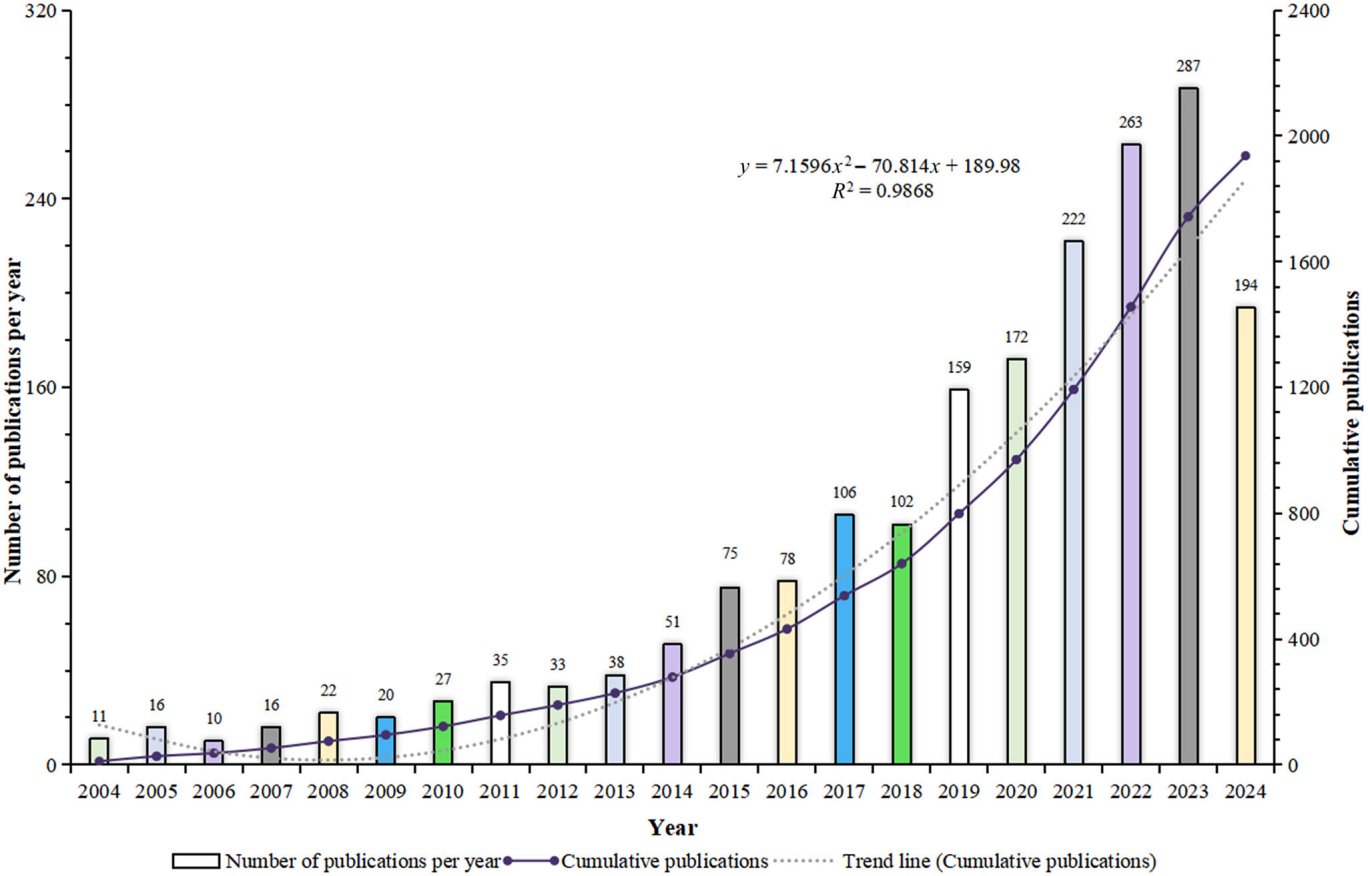
Annual published articles on postoperative delirium from 2004 to 2024.

The WoSCC database documented 1937 articles on POD published from January 1, 2004, to August 1, 2024 (see Figure 1). These articles spanned contributions from 65 countries/regions, 2148 institutions, and 10,110 authors. Figure 2 illustrates that the annual publication count has steadily risen since 2004. The 20‐year publication trajectory could be divided into three phases. From 2004 to 2014, growth was slow, averaging 25 articles per year, suggesting limited visibility for the field. From 2015 to 2018, publication output increased steadily, indicating growing interest in the field. After 2019, the number of articles rose sharply and peaked in 2023, reflecting intensified attention to POD research (*p* < 0.001, Monte Carlo permutation method).

### 3.1 Countries and Institutions

**FIGURE 3:**
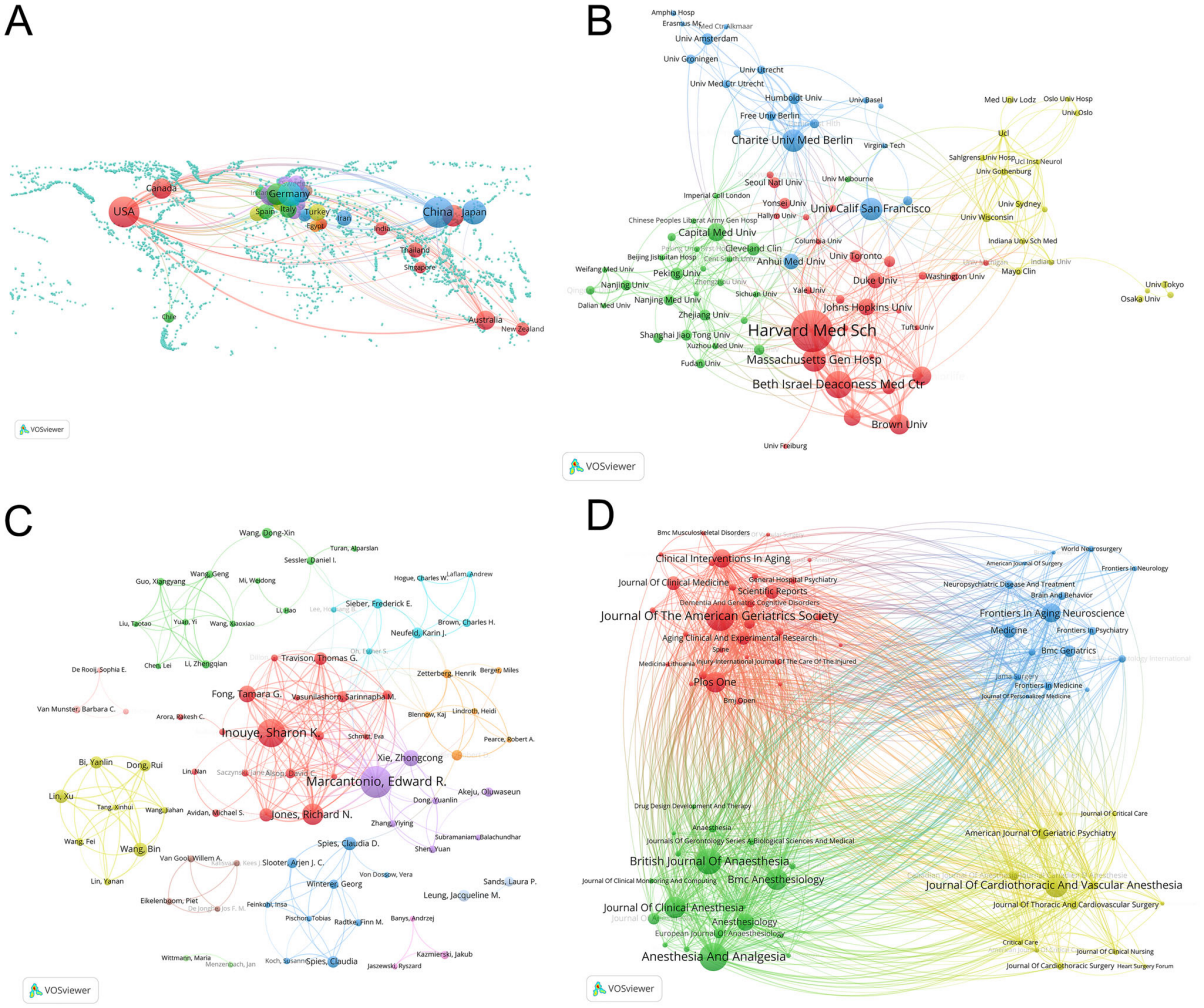
The network map of (A) countries, (B) institutions, (C) authors, and (D) journals.

Research on POD has been undertaken in 65 countries and regions. Table 3 details the yearly publication outputs of the 10 leading countries over the past two decades. China ranked first in publication volume (534 articles, 27.6%), followed closely by the United States (532 articles, 27.5%). In contrast, the United States had the highest total citations (20,204) and total link strength (285), whereas Canada had the highest citations per publication (41.9). These findings indicate that publication output, citation accumulation, and collaborative centrality were not fully aligned across countries. We then generated a co‐authorship map of 54 countries/regions with at least five publications (Figure 3A). The analysis showed the strongest collaboration between China and the United States, followed by Germany and the United States, and by Australia and the United States.

**TABLE 3:** Top 10 countries publishing literature related to postoperative delirium.

Rank	Country	Publications	Citations	Citations/publications	Total link strength^a^
1	China (Asia)	534	7468	13.985	114
2	United States (North America)	532	20,204	37.9774	285
3	Japan (Asia)	189	3093	16.3651	19
4	Germany (Europe)	185	4600	24.8649	136
5	South Korea (Asia)	109	1750	16.055	17
6	Netherlands (Europe)	108	3830	35.463	66
7	United Kingdom (Europe)	84	3040	36.1905	112
8	Canada (North America)	82	3437	41.9146	54
9	Australia (Oceania)	63	1161	18.4286	80
10	Italy (Europe)	40	1121	28.025	44

^a^ Total link strength refers to the cumulative strength of all links between a node and other nodes.

Table 4 shows that the 10 leading institutions accounted for 527 articles, or 27.2% of all publications. The three most productive institutions were Harvard Medical School (106 articles, 5.5%; USA), Beth Israel Deaconess Medical Center (64 articles, 3.3%; USA), and Massachusetts General Hospital (55 articles, 2.8%; USA). Although the Hebrew SeniorLife Institute for Aging Research ranked lower in total output, it achieved the highest average citation count (82.9), indicating broad recognition of its research impact. We also generated a clustering map of institutional collaborations using a minimum threshold of 10 documents per institution, as shown in Figure 3B. Harvard Medical School demonstrated the highest degree of collaboration with other institutions. The strongest institutional linkage was between Harvard Medical School and Beth Israel Deaconess Medical Center, as represented by the red cluster.

**TABLE 4:** Top 10 organizations on the research of postoperative delirium.

Rank	Organization	Publications	Citations	Citations/publications	Total link strength
1	Harvard Medical School (United States)	106	3921	36.9906	247
2	Beth Israel Deaconess Medical Center (United States)	64	3175	49.6094	201
3	Massachusetts General Hospital (United States)	55	1891	34.3818	155
4	Charité – Universitätsmedizin Berlin (Germany)	52	1204	23.1538	94
5	University of California, San Francisco (United States)	51	2443	47.902	45
6	Brown University (United States)	45	2048	45.5111	138
7	Hebrew SeniorLife (United States)	43	3563	82.8605	143
8	Capital Medical University (China)	39	554	14.2051	35
9	Johns Hopkins University (United States)	36	2102	58.3889	42
10	Brigham and Women's Hospital (United States)	36	1699	47.1944	102

### 3.2 Authors and Co‐Authors

Within POD research, Edward R. Marcantonio (USA) emerged as the most prolific author with 63 publications. Table 5 lists the 10 most productive authors in this field. The average citation count serves as an indicator of the scholarly recognition of an author's work. Of the top 10 authors, five averaged more than 50 citations per paper, and Richard N. Jones had the highest average citation count at 76.5. Furthermore, Table 5 lists the 10 most frequently co‐cited authors, each cited at least 200 times. The most frequently co‐cited author was Sharon K. Inouye (1165 citations), followed by Edward R. Marcantonio (587 citations) and E. Wesley Ely (496 citations).

**TABLE 5:** Top 10 authors and co‐cited authors on the research of postoperative delirium.

Rank	Author	Publications	Citations	Citations/publications	Co‐cited authors^a^	Total co‐citations
1	Marcantonio, Edward R. (United States)	63	3785	60.1	Inouye, Sharon K. (United States)	1165
2	Inouye, Sharon K. (United States)	54	3572	66.1	Marcantonio, Edward R. (United States)	587
3	Jones, Richard N. (United States)	39	2984	76.5	Ely, E Wesley. (United States)	496
4	Fong, Tamara G. (United States)	28	1773	63.3	Rudolph, James L. (United States)	430
5	Xie, Zhongcong (United States)	27	775	28.7	Saczynski, Jane S. (United States)	279
6	Wang, Bin (China)	23	92	4	Robinson, Thomas N. (United States)	268
7	Lin, Xu (China)	22	92	4.2	Aldecoa, César. (Spain)	223
8	Spies, Claudia D. (Germany)	22	400	18.2	Maldonado, Jose R. (United States)	222
9	Schmitt, Eva M. (United States)	22	1250	56.8	Witlox, Joost. (Netherlands)	216
10	Travison, Thomas G. (United States)	21	919	43.8	Folstein, M F. (United States)	209

^a^ Co‐cited authors were ranked from the top 20 prolific authors.

Analysis of the author collaboration network (minimum of eight documents per author; Figure 3C) showed active collaboration across clusters. The strongest collaboration was observed between Edward R. Marcantonio (purple cluster) and Sharon K. Inouye (red cluster). In addition, Sharon K. Inouye and Richard N. Jones were found to collaborate closely. The author time‐density map (Figure S1) showed that the three leading authors, including Edward R. Marcantonio, Sharon K. Inouye, and Richard N. Jones, published most actively during 2017–2019. Among the top 20 authors, Wang Bin, Lin Xu, and Bi Yanlin had a more recent average publication period (2022–2023), suggesting ongoing engagement with emerging topics in POD research.

### 3.3 Journals and Co‐Cited Journals

Table 6 lists the 10 journals with the highest publication output. These top 10 journals published 389 articles, accounting for 20.1% of total publications. Within this group, the *Journal of the American Geriatrics Society* led with 53 articles (2.7%), followed by *Anesthesia and Analgesia* with 51 articles (2.6%) and the *British Journal of Anaesthesia* with 47 articles (2.4%). All of these journals were classified as Q1 or Q2. *Anesthesiology* and the *British Journal of Anaesthesia* had the highest impact factors (IFs) among the journals analyzed, each with an IF of 9.1. Although *Anesthesiology* published fewer articles, it had one of the highest *H*‐indices. We further applied Bradford's law of scattering to classify journal productivity zones. The distribution of POD‐related publications was broadly consistent with the theoretical 1:*n*:*n* ^2^ Bradford pattern. The dataset comprised 1937 publications and was divided into three zones, each containing approximately equal numbers of articles. Zone 1 (the core zone) consisted of highly productive specialty journals, such as the *Journal of the American Geriatrics Society* and the *British Journal of Anaesthesia*, with a calculated Bradford multiplier (*k*) of approximately 4.8. A clustering analysis of 551 journals, each with at least six articles, was constructed based on publication output. Figure 3D illustrates strong co‐citation linkages among the *Journal of the American Geriatrics Society*, the *British Journal of Anaesthesia*, the *Journal of Cardiothoracic and Vascular Anesthesia*, and *Frontiers in Aging Neuroscience*.

**TABLE 6:** The top 10 journals with the most articles on postoperative delirium.

Rank	Journal	Impact factor (2023)	JCR	Counts	*H*‐index	Total link strength
1	*Journal of the American Geriatrics Society*	6.3	Q1	52	208	631
2	*Anesthesia And Analgesia*	4.6	Q1	51	187	554
3	*British Journal of Anaesthesia*	9.1	Q1	47	159	613
4	*Journal of Cardiothoracic and Vascular Anesthesia*	2.3	Q2	41	76	277
5	*BMC Anesthesiology*	2.3	Q2	37	31	284
6	*Journal of Clinical Anesthesia*	5	Q1	37	65	329
7	*PLOS One*	2.9	Q1	36	268	292
8	*Frontiers in Aging Neuroscience*	4.1	Q2	33	55	220
9	*Clinical Interventions in Aging*	3.6	Q2	32	59	266
10	*Anesthesiology*	9.1	Q1	29	214	432

Abbreviation: JCR, Journal Citation Reports.

**FIGURE 4:**
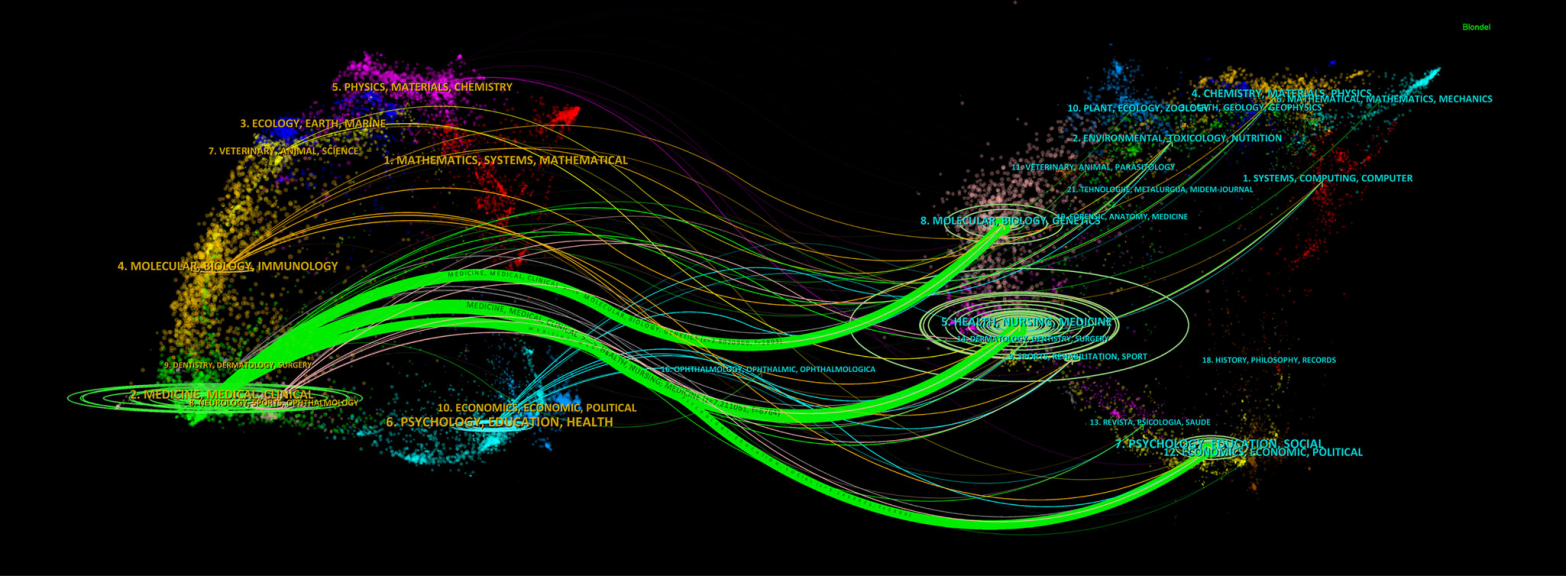
Dual‐map overlay of journals related to postoperative delirium. The citing journals are on the left, the cited journals are on the right, and the colored path represents the citation relationship.

Dual‐map overlay analysis provides insight into the evolution of disciplines and emerging scientific frontiers. Figure 4 displays a dual‐map overlay visualization for articles on POD research. On the dual map, journals on the left denote applied disciplines, whereas those on the right reflect fundamental research areas. The curves depict citation trajectories within the map. Publications on POD predominantly appeared in journals centered on medical and clinical disciplines. Citations mainly originated from journals covering molecular biology, genetics, health, nursing, medicine, psychology, education, and the social sciences. The dual‐map overlay suggests increased bibliometric attention to research linking molecular mechanisms to clinical applications.

### 3.4 Knowledge Base and Highly Co‐Cited References

Table 7 lists the 15 most frequently co‐cited references in POD research over the past two decades, many of which represent foundational works in the field. Of these references, four were review articles addressing the epidemiology, clinical features, and management strategies of POD. There were evidence‐based consensus guidelines on POD. Six focused on perioperative and POD risk and prevention, including clinical studies and meta‐analyses. Two studies are cohort analyses investigating the link between delirium and cognitive trajectories. Several of these represent international collaborative efforts. Most of these references were published in high‐impact, Q1 journals, underscoring their visibility within the field. The 2014 review by Inouye SK et al. had the highest co‐citation frequency (153 citations) and the highest IF among these references (98.4; *Lancet*). This review synthesized the epidemiology, clinical features, and management of delirium in older adults and has been highly influential in the field.

**TABLE 7:** Top 15 most co‐cited references in postoperative delirium.

Rank	Title	Year	Journal	IF (2023)	JCR	Author	Citations	PubMed ID
1	Delirium in elderly people	2014	*Lancet*	98.4	Q1	Inouye SK	153	23992774
2	European Society of Anaesthesiology evidence‐based and consensus‐based guideline on postoperative delirium	2017	*European Journal of Anaesthesiology*	4.2	Q1	Aldecoa C	141	28187050
3	Postoperative delirium: Perioperative assessment, risk reduction, and management	2020	*British Journal of Anaesthesia*	9.1	Q1	Jin ZS	106	32798069
4	Cognitive trajectories after postoperative delirium	2012	*New England Journal of Medicine*	96.3	Q1	Saczynski JS	76	22762316
5	Delirium in hospitalized older adults	2017	*New England Journal of Medicine*	96.3	Q1	Marcantonio ER	57	29020579
6	Delirium	2020	*Nature Reviews Disease Primers*	79.0	Q1	Wilson JE	56	33184265
7	The short‐term and long‐term relationship between delirium and cognitive trajectory in older surgical patients	2016	*Alzheimer's & Dementia*	13.1	Q1	Inouye SK	46	27103261
8	Dexmedetomidine for prevention of delirium in elderly patients after non‐cardiac surgery: A randomised, double‐blind, placebo‐controlled trial	2016	*Lancet*	98.4	Q1	Su X	46	27542303
9	Risk factors for postoperative delirium following hip fracture repair in elderly patients: A systematic review and meta‐analysis	2017	*Aging Clinical and Experimental Research*	3.4	Q1	Yang YJ	46	26873816
10	American Society for Enhanced Recovery and Perioperative Quality Initiative Joint Consensus Statement on Postoperative Delirium Prevention	2020	*Anesthesia & Analgesia*	4.6	Q1	Hughes CG	45	32022748
11	Prevention of postoperative delirium in elderly patients planned for elective surgery: Systematic review and meta‐analysis	2019	*Clinical Interventions in Aging*	3.5	Q2	Janssen TL	44	31354253
12	Risk factors of postoperative delirium after cardiac surgery: A meta‐analysis	2021	*Journal of Cardiothoracic Surgery*	1.5	Q3	Chen HY	43	33902644
13	Recommendations for the nomenclature of cognitive change associated with anaesthesia and surgery‐2018	2018	*British Journal of Anaesthesia*	9.1	Q1	Evered L	41	30336844
14	Effect of delirium and other major complications on outcomes after elective surgery in older adults	2015	*JAMA Surgery*	15.9	Q1	Gleason LJ	38	26352694
15	Delirium in older persons: Advances in diagnosis and treatment	2017	*JAMA ‐ The Journal of the American Medical Association*	63.5	Q1	Oh ES	38	28973626

**FIGURE 5:**
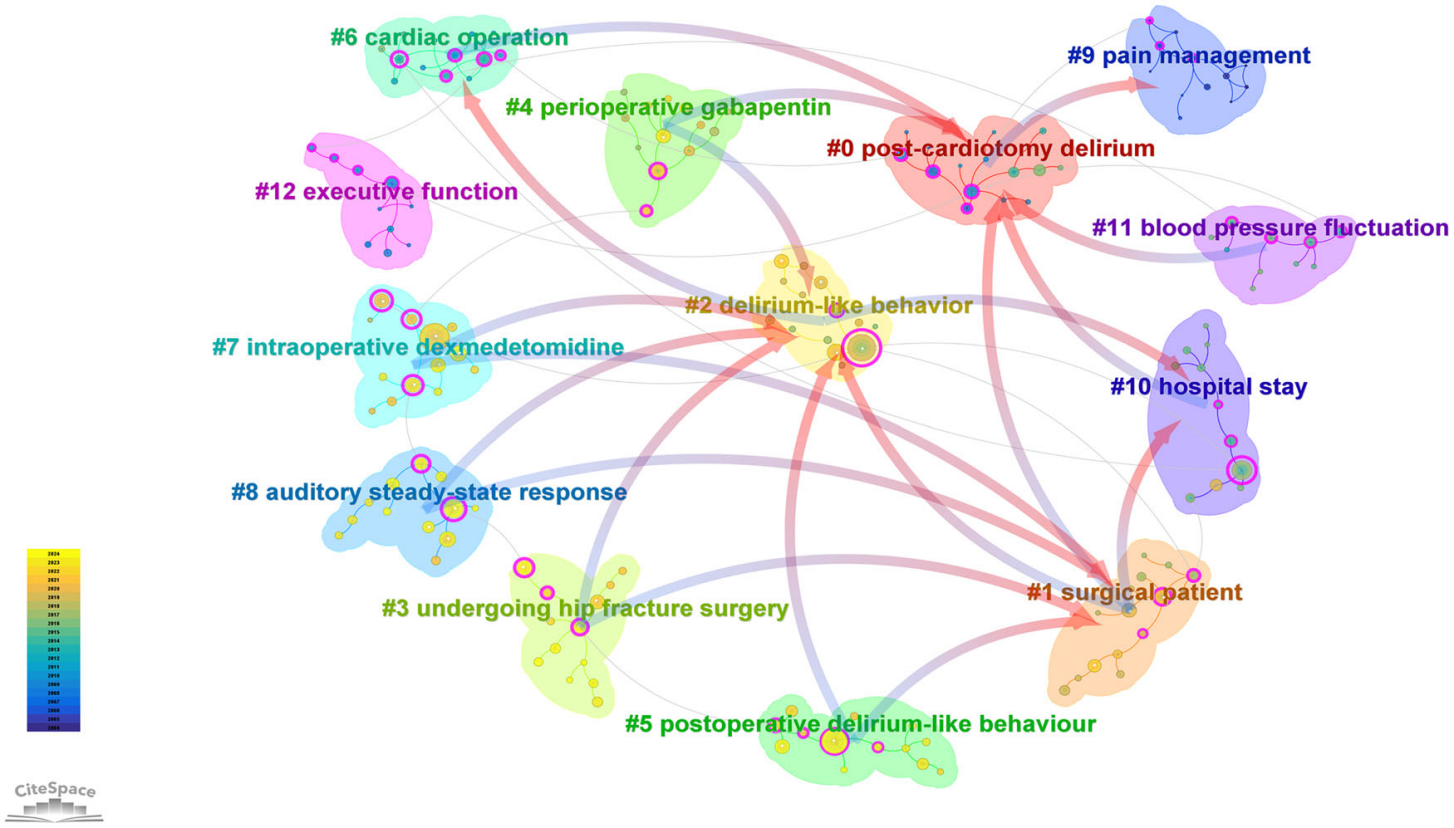
Co‐citation clustering map for postoperative delirium research.

A co‐citation clustering map for POD research was also generated (see Figure 5). CiteSpace was configured with the following parameters: time slicing from 2004 to 2024, 1‐year slices, and a selection criterion *k* = 2. Overlapping circle size reflects total annual citation counts: purple denotes earlier citations, yellow denotes more recent citations, and mixed hues represent citations spanning multiple years. Ultimately, the co‐cited references were organized into 13 clusters, namely: #0 post‐cardiotomy delirium, #1 surgical patient, #2 delirium‐like behavior, #3 hip fracture surgery, #4 perioperative gabapentin, #5 postoperative delirium‐like behavior, #6 cardiac operation, #7 intraoperative dexmedetomidine, #8 auditory steady‐state response, #9 pain management, #10 hospital stay, #11 blood pressure fluctuation, and #12 executive function. Research related to delirium‐like behavior has shown increased recent citation activity.

### 3.5 Keywords

**FIGURE 6:**
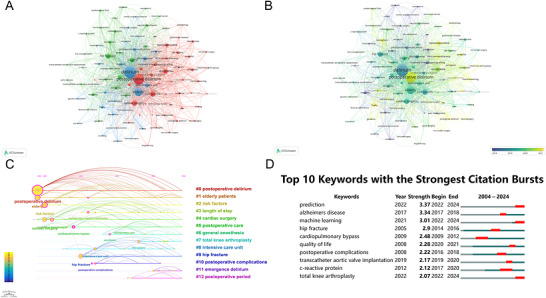
The (A) network, (B) overlay, and (C) timeline map of keyword co‐occurrence. (D) The top 10 keywords with the strongest citation bursts.

Keyword analysis provides a rapid overview of a field and its evolving trends. Table 8 lists the 20 most frequently occurring keywords, with “delirium” (733 occurrences) leading, followed by “postoperative delirium” (589), “elderly” (292), “risk factors” (155), and “cardiac surgery” (141). Co‐occurrence analysis of keywords is a standard method for pinpointing prevalent research themes. Figure 6A,B displays the network and overlay visualizations of co‐occurring keywords (with a minimum occurrence threshold of 8). In Figure 6A, keywords are partitioned into three distinct clusters. The largest red‐colored cluster focused on surgical and anesthetic topics, including “postoperative delirium,” “risk factors,” “cardiac surgery,” “anesthesia,” and “dexmedetomidine.” The green cluster, the second largest, focused on hip fracture surgery and neuroinflammatory markers, including “hip fracture,” “cognitive impairment,” “surgery,” “inflammation,” and “biomarkers.” The blue cluster was associated with delirium in older patients in intensive care units (ICUs) and included “delirium,” “elderly,” “ICU,” and “postoperative complications.”

**TABLE 8:** The top 20 keywords for postoperative delirium.

Rank	Keyword	Counts
1	Delirium	733
2	Postoperative delirium	589
3	Elderly	292
4	Risk factors	155
5	Cardiac surgery	141
6	Hip fracture	135
7	Anesthesia	106
8	Surgery	99
9	Cognitive impairment	86
10	Intensive care unit	68
11	Postoperative	59
12	Dexmedetomidine	58
13	Coronary artery bypass grafting	46
14	Dementia	46
15	Cardiopulmonary bypass	44
16	Postoperative complications	43
17	Cognitive assessment screening instrument	42
18	Inflammation	41
19	Prediction	41
20	Mortality	40

Figure 6B indicates that blue‐colored terms correspond to an average publication year of 2018 or earlier, whereas bright yellow terms have appeared post‐2020. Keywords like “coronary artery bypass grafting,” “interleukin‐6,” “postoperative complications,” and “mortality” dominated the earlier period. Conversely, terms such as “dexmedetomidine,” “biomarkers,” “frailty,” and “machine learning” have surfaced more recently. Additionally, CiteSpace was used to visualize the temporal evolution of keywords (see Figure 6C). This visualization yielded a *Q* value of 0.8593 and an *S* value of 0.9688. Before 2010, “postoperative delirium” and “elderly” emerged as the principal hotspot keywords. Projections for 2024 suggest that “postoperative delirium,” “risk factors,” “length of stay,” “elderly patients,” and “general anesthesia” will continue as prominent topics. Another key metric for identifying research frontiers and hotspots over time is keyword burst strength (see Figure 6D). Among the 10 keywords exhibiting the highest citation burst strength, “prediction” showed the most robust burst (3.37), followed by “Alzheimer's disease” (3.34) and “machine learning” (3.01). Notably, “prediction,” “machine learning,” and “total knee arthroplasty” experienced burst activity in 2024, indicating ongoing research attention in these areas.

### 3.6 Possible Biomarkers and Diseases

**FIGURE 7:**
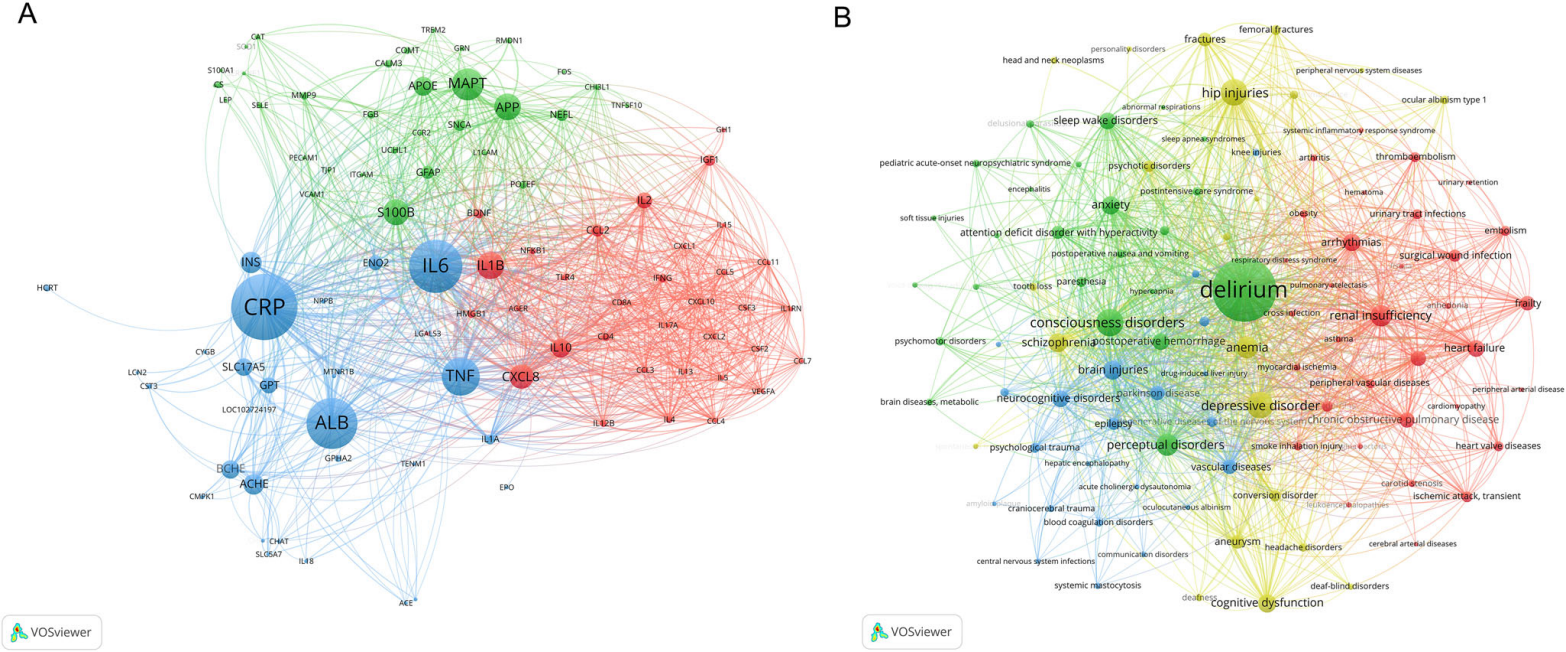
Co‐occurrence network analysis of (A) biomarkers and (B) diseases associated with postoperative delirium.

Using VOSviewer, we performed co‐occurrence clustering of potential biomarkers and diseases associated with POD. In total, 494 biomarkers and 1127 diseases were extracted from 1937 articles, as shown in Figure 7A. Within the blue cluster, C‐reactive protein (CRP), albumin, interleukin 6 (IL‐6), and tumor necrosis factor (TNF) were most prominent. Within the green cluster, microtubule‐associated protein tau, amyloid precursor protein, apolipoprotein E (APOE), and S100B predominated, whereas IL1B and C‐X‐C motif chemokine ligand 8 (CXCL8) were most frequently discussed in the red cluster. These biomarkers are broadly associated with inflammatory pathways or neurodegenerative processes, supporting their relevance in the POD literature. Figure 7B shows that renal insufficiency, heart failure, disorders of consciousness, and brain and hip injuries were recurrently co‐mentioned disease terms within the POD literature. These patterns suggest areas of sustained clinical attention rather than direct causal relationships.

## 4 Discussion

### 4.1 Summary of Main Findings

This study provides a bibliometric overview of POD research over the past two decades and helps clarify the field's development over time. Beyond documenting growth in publication volume, the findings show that influence in this field is unevenly distributed across output, citation accumulation, and collaboration. Publication activity accelerated sharply after 2019, but the countries and institutions that contributed the most papers were not always those with the greatest citation visibility or network centrality. This pattern suggests that POD research has entered a stage of rapid expansion in which productivity, scholarly recognition, and international integration are developing at different rates.

Although China ranked first in publication volume, its citations per publication were lower than those of several Western countries, particularly the United States and Canada. This divergence should be interpreted cautiously and is unlikely to reflect scientific quality alone. First, citation lag probably contributed, because publication output in this field accelerated markedly after 2019, and newer articles have had less time to accumulate citations. Second, the United States occupied the most central position in the collaboration network, with substantially higher total link strength than China, which may have enhanced international visibility and downstream citation uptake. Third, many of the field's most productive and highly cited authors and institutions, including Edward R. Marcantonio, Sharon K. Inouye, Richard N. Jones, Harvard Medical School, and Beth Israel Deaconess Medical Center, were based in the United States, suggesting that earlier‐established research hubs continue to shape the citation structure of the field. Therefore, citation‐based influence in POD research appears to be determined not only by output volume but also by publication timing, network centrality, and historical leadership within the field.

Identifying research hotspots and frontiers is essential for understanding the field's evolution. After integrating co‐citation clusters with keywords showing strong burst activity, four major thematic domains were identified: (1) characteristics, (2) mechanisms, (3) prediction, and (4) interventions. Collectively, these domains underscore the multifaceted nature of POD and highlight diverse avenues for improving its detection and management.

### 4.2 Characteristics

The “characteristics” domain reflects a persistent focus in the POD literature on cognitive vulnerability, postoperative cognitive trajectories, and the interface between delirium and dementia. In our bibliometric analysis, this domain was supported by the prominence of keywords related to cognitive impairment, elderly patients, executive function, and Alzheimer's disease, as well as by highly co‐cited references addressing delirium phenotypes, diagnostic boundaries, and long‐term cognitive outcomes (Fong et al. 2015; Needham et al. 2017; American Psychiatric Association 2013; Fong and Inouye 2022). These patterns suggest that a substantial portion of the field has been concerned not merely with POD as an acute perioperative event but with how it fits within broader trajectories of brain aging and neurocognitive decline.

A recurring theme in this domain is the distinction and overlap between delirium and dementia. Highly cited conceptual work has emphasized that delirium is an acute and fluctuating neurocognitive syndrome, whereas dementia is typically progressive and chronic; however, the two conditions are closely interrelated in older adults (Fong et al. 2015; Needham et al. 2017; American Psychiatric Association 2013; Fong and Inouye 2022). The continued visibility of these references in the co‐citation network suggests that clarifying this relationship remains foundational to POD scholarship. This is further supported by the sustained appearance of terms such as “elderly,” “cognitive impairment,” and “executive function” in the keyword maps, indicating that patient susceptibility and baseline cognitive reserve remain central to how POD is framed in the literature.

Another characteristic feature of this domain is the growing emphasis on postoperative cognitive trajectories rather than isolated delirium episodes. Several influential cohort studies have shown that delirium is associated with acute decline followed by incomplete recovery and, in some patients, subsequent long‐term deterioration (Goldberg et al. 2020; Kunicki et al. 2023; McKeith et al. 2020; Inouye et al. 2016; Richardson et al. 2021). From a bibliometric perspective, the prominence of these studies suggests that the field is increasingly attentive to POD as a marker of broader neurocognitive vulnerability rather than merely a transient perioperative complication. This interpretive shift is important because it expands the clinical significance of POD from short‐term symptom recognition to longer‐term functional and cognitive outcomes (Jackson et al. 2015; Goldberg et al. 2020; Kunicki et al. 2023; McKeith et al. 2020; Inouye et al. 2016; Richardson et al. 2021; Megari and Kosmidis 2024).

The frequent co‐occurrence of Alzheimer‐related terms also indicates sustained interest in the overlap between POD and neurodegenerative disease. In particular, the bibliometric prominence of Alzheimer's disease, tau‐related biomarkers, and APOE suggests that the literature increasingly considers POD within a framework of latent neurodegenerative susceptibility (Lopez and Kuller 2019; Janelidze et al. 2021; Thijssen et al. 2021; Liang et al. 2023). These patterns do not establish a direct causal continuum between delirium and dementia. However, they do indicate that the field is moving toward a more integrated conceptualization in which acute perioperative delirium, preexisting cognitive reserve, and neurodegenerative biology are studied in relation to one another. Overall, the characteristics domain shows that POD research has progressively evolved from syndrome description toward characterization of vulnerable cognitive phenotypes and longer‐term neurocognitive trajectories.

### 4.3 Mechanisms

The “mechanisms” domain reflects how the POD literature has clustered around several recurring biological themes rather than a single unified pathogenic model. In our bibliometric analysis, the biomarker co‐occurrence map (Figure 7A) showed prominent clustering of inflammatory mediators, including CRP, IL‐6, TNF, IL1B, and CXCL8, as well as neurodegeneration‐related proteins such as tau, amyloid precursor protein, APOE, and S100B. These patterns suggest that the mechanistic literature on POD has been shaped primarily by two intersecting lines of inquiry: systemic and neuroinflammatory signaling, and the overlap between delirium vulnerability and neurodegenerative biology.

The predominance of inflammatory markers in the co‐occurrence network suggests sustained bibliometric attention to neuroinflammation as a central explanatory theme in POD research. Rather than establishing neuroinflammation as the single definitive mechanism, this pattern suggests that inflammatory signaling has become the most prominent mechanistic framework for studying perioperative brain dysfunction (Subramaniyan and Terrando 2019; Hoogland et al. 2015). Similarly, the frequent appearance of tau‐, APOE‐, and amyloid‐related terms suggests growing interest in the relationship between POD and neurodegenerative susceptibility, especially in older surgical populations (Alam et al. 2018; Zhang et al. 2022; Murray et al. 2012; Jia et al. 2024; Heinrich et al. 2021; Adam et al. 2020).

Additional themes, including extracellular vesicles (EVs), mitochondrial dysfunction, and the gut–brain axis, appear to represent more recent and expanding areas of mechanistic exploration (Valotto Neto et al. 2024; Hotta et al. 2022; Cho et al. 2024). In bibliometric terms, these topics may be interpreted as emerging subthemes within the broader mechanisms domain, reflecting increasing interest in circulating biomarkers, metabolic vulnerability, and host–microbiome interactions. However, their current prominence in the literature should be interpreted as evidence of thematic growth rather than confirmation of mechanistic primacy or clinical applicability (Liu et al. 2022; W. Yu, Zhu, et al. 2024; Lu et al. 2022; Liufu et al. 2020).

Overall, the bibliometric structure of the mechanisms domain suggests that POD research is moving toward a more biomarker‐oriented and biologically stratified understanding of perioperative neurocognitive vulnerability. Importantly, these patterns reflect the distribution of research attention within the literature rather than direct proof of causality or relative mechanistic importance.

### 4.4 Prediction

The “prediction” domain captures a major shift in the POD literature toward identifying patients at high risk before overt clinical manifestations occur. In our bibliometric analysis, this domain was supported by the strongest keyword burst for “prediction,” the recent visibility of “machine learning,” and co‐citation clusters related to blood pressure fluctuation, postoperative complications, and surgical risk contexts. The emergence of “prediction,” “machine learning,” and Alzheimer‐related biomarker terms suggests that the field is moving from descriptive characterization of delirium toward anticipatory risk stratification and biologically informed perioperative phenotyping.

Identifying reliable biomarkers for POD is essential for timely diagnosis and intervention. Recent research has identified several potential biomarkers, including inflammatory cytokines, neurotrophic factors, and genetic predispositions. Among the identified pathogenic mechanisms, elevated levels of TNF‐α, IL‐1β, IL‐6, IL‐8, IL‐10, CRP, S100B, matrix metalloproteinase‐9, brain‐derived neurotrophic factor, copeptin, and cortisol, as well as the presence of the APOE ε4 allele, have been associated with a higher risk of POD (Fournier et al. 2015; van den Boogaard et al. 2011). A recent study suggests that tau‐based plasma proteins may serve as risk biomarkers for POD (Thijssen et al. 2021). This observation suggests that disorders sharing pathogenic mechanisms with delirium may also share biomarkers, which could guide future biomarker discovery.

In addition to biological markers, machine learning algorithms have emerged as potent tools for predicting POD. By analyzing preoperative, intraoperative, and postoperative data, these algorithms can identify patterns and risk factors that may be missed by conventional clinical assessment. For example, POD incidence varies by surgical type, with the overall rate in the elderly ranging from 15% to 25% and rising to 50% following high‐risk procedures such as hip fracture repair or cardiac surgery (Gleason et al. 2015; C.‐G. Wang et al. 2018). Although several studies and a recent meta‐analysis reported no association between intraoperative hypotension and delirium (Maheshwari et al. 2020; Wesselink et al. 2018), other findings indicate that POD is associated with increased intraoperative vasopressor use (Neerland et al. 2017; Rudiger et al. 2016). This suggests that incorporating surgical types, intraoperative blood pressure variations, and vasopressor administration into predictive models may improve the forecasting of POD. Models incorporating demographic data, comorbidities, and surgical variables have shown promise in accurately predicting the onset of delirium and other postoperative complications (Lee et al. 2023; Yoshimura et al. 2024). Importantly, the bibliometric prominence of machine learning‐related terms should be interpreted as evidence of increasing scholarly attention rather than proof of clinical maturity, external validity, or implementation readiness.

Moreover, studies indicate that the performance of various models differs (Yang et al. 2024; Xue et al. 2021). Despite promising results, important challenges remain. Most machine learning models in the POD literature are assessed as having a notably high risk of bias using tools such as the PROBAST (Prediction Model Risk of Bias Assessment Tool). The algorithms in use are not ready for implementation in clinical settings due to limitations such as inadequate handling of missing data, severe class imbalance (e.g., hyperactive‐agitated delirium is much rarer than elusive‐hypoactive delirium), uninterpretable “black box” algorithm designs, and alarmingly low events‐per‐variable ratios.

### 4.5 Interventions

The “interventions” domain encompasses both pharmacological and nonpharmacological strategies to prevent POD and mitigate its downstream consequences. However, these approaches do not share identical evidence structures. Pharmacological interventions are generally easier to evaluate in masked randomized trials, whereas nonpharmacological interventions are typically delivered as multicomponent care bundles and are more often supported by prospective implementation studies, pragmatic clinical programs, and consensus recommendations.

Among pharmacological strategies, dexmedetomidine appears to have the most consistent support. Randomized trials, meta‐analyses, and consensus statements suggest that it may reduce POD risk in selected perioperative settings, particularly in older adults undergoing major noncardiac surgery (Zeng et al. 2016; Flükiger et al. 2018; K. Wang et al. 2019). Nevertheless, heterogeneity in dose, timing, and patient selection continues to limit universal adoption.

By contrast, other medications show weaker or inconsistent support. Gabapentin has attracted substantial research attention, but higher‐level evidence remains unconvincing: randomized studies did not demonstrate a reduction in POD incidence or hospital stay, and more recent analyses have raised concern regarding a possible increase in delirium risk among vulnerable older adults. Likewise, evidence for antipsychotics remains inconclusive and is constrained by adverse‐effect concerns, including cardiometabolic toxicity and drug interactions (Leung et al. 2006; Leung et al. 2017; Oh et al. 2019). Accordingly, the pharmacological evidence base is internally heterogeneous, and dexmedetomidine is the only agent discussed here with comparatively consistent support across multiple evidence tiers (León‐Salas et al. 2020).

Pain management occupies an intermediate position in this framework. Effective multimodal analgesia is clinically important because uncontrolled pain may contribute to delirium vulnerability, but pain control strategies should be understood primarily as supportive risk‐modifying measures rather than as stand‐alone delirium‐specific therapies (Sica et al. 2023).

Nonpharmacological interventions should be regarded as the foundational component of POD prevention and management. Their importance is supported by major delirium guidelines and influential multicomponent care programs such as the Hospital Elder Life Program (HELP; Ma et al. 2023). These approaches emphasize orientation, early mobilization, sleep optimization, sensory support, and multidisciplinary coordination. Although fewer large, blinded, randomized trials exist in this domain, this largely reflects the methodological difficulty of testing complex system‐level interventions rather than a lack of clinical relevance (L. Yu, Yang, et al. 2024; Wilson et al. 2020; Inouye et al. 1999).

Overall, the hierarchy of evidence in this field suggests that nonpharmacological multicomponent care remains the basis of prevention, whereas pharmacological measures should generally be viewed as adjunctive and context specific. Among medications, dexmedetomidine currently has the strongest supporting evidence, while the benefits of other agents remain limited or uncertain.

### 4.6 Limitations

Bibliometric analyses have several inherent limitations. First, citation frequency reflects scholarly visibility rather than methodological rigor. Highly cited POD studies may still be limited by small sample sizes, heterogeneous diagnostic approaches, geographic publication bias, or unequal research resources. Second, although recent studies commonly use validated screening tools such as the Confusion Assessment Method (CAM) and CAM‐ICU, many earlier but still influential studies relied on retrospective chart review or inconsistent application of Diagnostic and Statistical Manual of Mental Disorders, Fourth Edition (DSM‐IV) or Diagnostic and Statistical Manual of Mental Disorders, Fifth Edition (DSM‐5) criteria. Third, short follow‐up periods, such as assessment only through postoperative day 3, may fail to capture delayed delirium and subsequent cognitive decline, potentially leading to underestimation of incidence and related economic burden. Fourth, bibliometric studies are vulnerable to publication bias. Positive studies, particularly those evaluating pharmacologic interventions such as dexmedetomidine, may be overrepresented in citation networks, whereas robust trials reporting null findings may be less visible. Fifth, bibliometric methods emphasize quantitative measures, such as publication output and citation counts, but offer limited insight into study quality, clinical relevance, or real‐world impact. Sixth, restriction to English‐language publications may underrepresent relevant work from non‐English‐speaking regions and may influence apparent geographic and thematic patterns. Seventh, newer publications, authors, and institutions may appear less influential simply because they have had less time to accumulate citations. Finally, burst keywords, overlay maps, and co‐occurrence clusters should be interpreted as indicators of shifting research attention rather than definitive evidence of paradigm change. Terms such as “emerging” or “growing” therefore require confirmation through focused systematic reviews and high‐quality primary studies. To address these limitations, future work may benefit from a hybrid “research weaving” approach that combines bibliometric mapping with PRISMA‐guided systematic review. Such an approach would allow broad visualization of the field while enabling more rigorous evaluation of clinical effectiveness within specific thematic areas.

## 5 Conclusion

This bibliometric analysis provides a comprehensive overview of POD research over the past two decades and identifies major thematic and collaborative patterns in the literature. The findings suggest that cardiac and orthopedic surgery, blood pressure fluctuations, neuroinflammation, cognitive vulnerability, biomarker discovery, and predictive modeling have received sustained attention within the field. In particular, prediction‐related and biomarker‐related topics have become increasingly prominent in recent years. These bibliometric signals may help inform future research priorities, but they should be interpreted alongside high‐quality clinical and mechanistic evidence.

## Author Contributions

**Kehan Wang**: writing – original draft, methodology, investigation, formal analysis, conceptualization. **Cheng Xu**: writing – original draft, validation, software, resources, project administration, methodology, investigation, funding acquisition, data curation, conceptualization. **Yuan Zhu**: writing – original draft, methodology, investigation, funding acquisition, data curation, conceptualization. **Quanhong Zhou**: writing – review and editing, methodology, investigation. **Tao Xu**: writing – review and editing, visualization, validation, supervision, software, resources, project administration, methodology, investigation, funding acquisition, conceptualization.

## Funding

This study was supported by grants from the National Natural Science Foundation of China (NSFC) (82525022, 82371284, including the Young Scientists Fund, Category A), the National Key R&D Program of China (2024YFA1306902), the Research Physician Program of Shanghai Jiao Tong University School of Medicine (20240814), the Shanghai Eastern Talent PlanLeading Talent Project (BJWS2024040), and High‐level Talent Support and Cultivation Program of Shanghai Sixth People's Hospital (ynljzc202602).

## Ethics Statement

The authors have nothing to report.

## Conflicts of Interest

The authors declare no conflicts of interest.

## Supporting information



Figure 1. The author time density map for postoperative delirium research

## Data Availability

The data that support the findings of this study are available from the corresponding author upon reasonable request.
