# Supplementary material for: Evolving Trends and Collaborative Networks in Postoperative Delirium Research: A Two‐Decade Bibliometric Analysis
Source: Brain Behav. 2026 Jun 22;16(6):e71558. doi: 10.1002/brb3.71558 (PMC13284738; doi:10.1002/brb3.71558)
Supplement: Supplementary file 1 — Figure 1. The author time density map for postoperative delirium research [file BRB3-16-e71558-s001.docx]

**Supplementary Material**

**Supplementary Figure 1:**


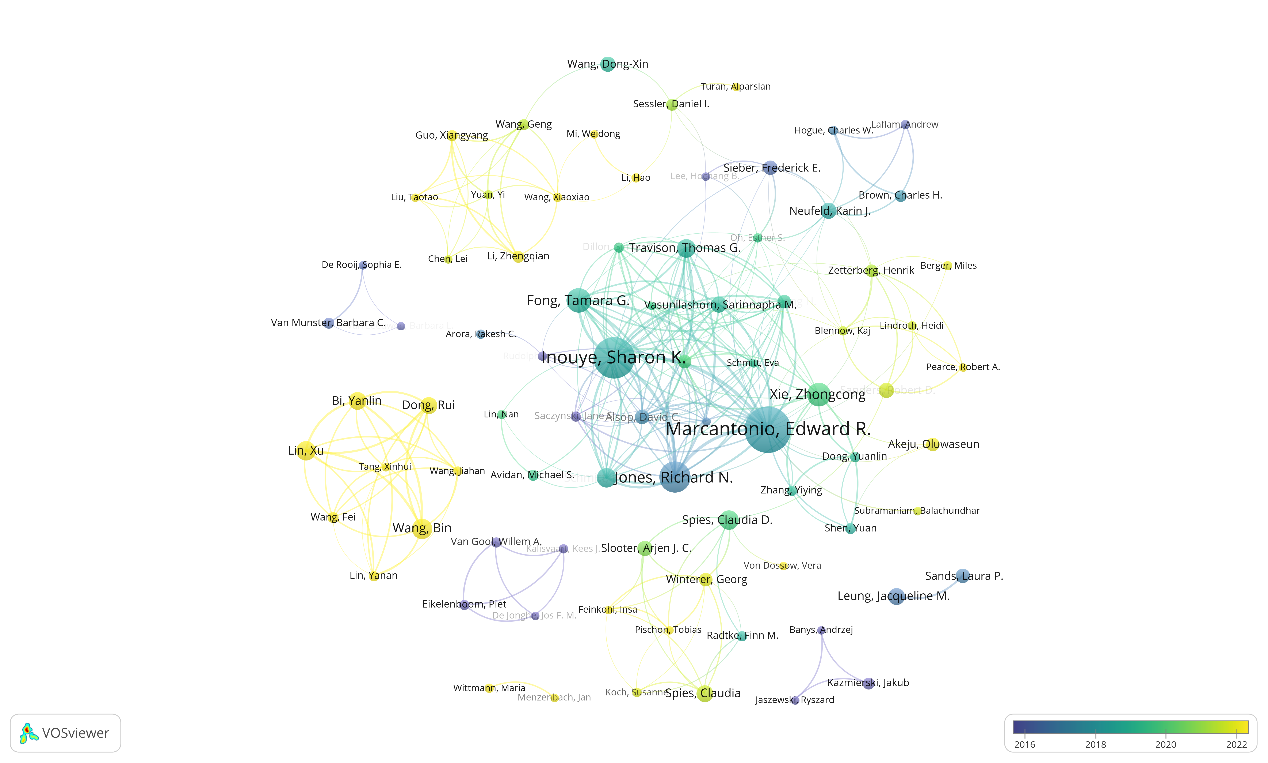


**Figure 1. The author time density map for postoperative delirium research**
